# Ixazomib‐based maintenance therapy after bortezomib‐based induction in patients with multiple myeloma not undergoing transplantation: A real‐world study

**DOI:** 10.1002/cam4.4313

**Published:** 2021-10-16

**Authors:** Man Shen, Jiajia Zhang, Ran Tang, Yuhao Wang, Xiaokai Zhan, Sibin Fan, Zhongxia Huang, Yuping Zhong, Xin Li

**Affiliations:** ^1^ Department of Hematology Multiple Myeloma Medical Center of Beijing Beijing Chao‐yang Hospital Capital Medical University Beijing China; ^2^ Department of Hematology Qingdao Municipal Hospital School of Medicine Qingdao University Qingdao China

**Keywords:** bortezomib, ixazomib, maintenance therapy, multiple myeloma, not undergoing transplantation, real‐world

## Abstract

**Background:**

Maintenance therapy with proteasome inhibitors (PIs) can improve outcomes of multiple myeloma (MM) patients, however, the neurotoxicity and parenteral route of bortezomib limit its long‐term use. An efficacious, tolerable, and convenient PI option is needed.

**Methods:**

In this single‐center, real‐world study, we retrospectively analyzed the outcome and safety profile of ixazomib‐based maintenance therapy in patients who plateaued with the responses of steady disease or better after bortezomib‐based induction therapy in MM patients not undergoing transplantation.

**Results:**

Of all the 71 patients, 37 cases (52.1%) were newly diagnosed MM (NDMM) and 34 cases (47.9%) were relapsed and/or refractory MM (RRMM). The overall response rate (ORR) was 81.7%, including 34 patients (47.9%) with a very good response rate or better (≥VGPR) after a median of nine cycles (6–14) of bortezomib‐based induction therapy. Then the ORR was transformed to 74.6% including 39 patients of ≥VGPR (54.9%) after a median of six courses (2–25) of ixazomib‐based maintenance therapy. Of these, 18 patients (25.4%) exhibited responses deepened. With 26.5 months median follow‐up, median progression‐free survival (PFS) was 28.4 and 16.5 months from the start of bortezomib and 16.2 and 10.0 months from the initiation of ixazomib in NDMM and RRMM group, respectively. Moreover, responses deepened during the maintenance phase (hazard ratio: 0.270, *p *= 0.007), and responses of ≥VGPR during the induction phase (hazard ratio: 0.218, *p *< 0.001) were confirmed to independently predict longer PFS after multivariate analyses. Severe adverse events (grade 3/4) were relatively rare. Bortezomib‐emergent peripheral neuritis (PN) was significantly relived after the transition to ixazomib (*p *< 0.001).

**Conclusion:**

This real‐world analysis has demonstrated oral ixazomib is a favorable option of long‐term administration for maintenance with efficacy and feasibility and confirmed the association between deepening responses with ixazomib and prolonged PFS.

## Graphical abstract

In real‐world MM population, ixazomib‐based regimen is a good choice of maintenance therapy for patients not undergoing transplantation, with efficiency, safety and convenience. Besides, deepened responses during maintanence may bring benefits to PFS.

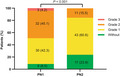

## 1 INTRODUCTION

Multiple myeloma (MM) is a hematological malignancy with a median survival of at least ten years, characterized by the accumulation of plasma cells in the bone marrow, which results in renal injury, osteolysis, hypercalcium, and anemia.^1^ Despite improvement in the prognosis of MM was observed with the increasing application of novel agents such as proteasome inhibitors (PI) and immunomodulatory drugs (Imids) over the past two decades, the disease remains largely incurable, with almost all patients eventually relapsing.^2^

Presently, the paradigm of long‐term therapy in MM has been widely accepted, since continuous or maintenance therapy after plateau demonstrated obvious prolonged disease control.^3^, ^4^ For not undergoing transplantation patients, long‐term bortezomib‐based therapy has improved progression‐free survival (PFS) and overall survival (OS) in global clinical trials,^5^, ^6^, ^7^ compared with fixed‐duration therapy. However, the risk of peripheral neuropathy (PN) and parenteral administration limits its long‐term use.^8^, ^9^ There remains a need for a tolerable, efficacious, and convenient PI option for long‐term maintenance therapy.

Ixazomib is the first oral PI approved for the treatment in MM patients who have received at least one previous therapy in over 60 countries. In the global phase Ⅲ TOURMALINE‐MM1 study and China Continuation Study (CCS),^10^, ^11^ the all‐oral combination of weekly ixazomib plus lenalidomide and dexamethasone (IRd) has demonstrated prolonged PFS with good safety and tolerability in relapsed and/or refractory (RR) MM.^10^, ^12^ Similar results were shown in global phase Ⅰ/Ⅱ clinical trials of newly diagnosed (ND) MM and a phase Ⅲ clinical trial of maintenance treatment of MM patients.^12^, ^13^ Recently, a real‐world multi‐center study from China reported that ixazomib‐based frontline therapy in NDMM showed comparable efficacy and safety profile with clinical trials.^14^ Nevertheless, there was limited report about the use of ixazomib‐based regimen as maintenance therapy in patients not undergoing transplantation in China.

The study aims to retrospectively evaluate the efficacy and safety of ixazomib as maintenance therapy in MM patients who plateaued after bortezomib‐based regimens in the real‐world setting.

## 2 METHODS

### 2.1 Patients

This single‐institutional, retrospective, observational study was conducted in Beijing Chao‐yang hospital. A total of 118 patients with NDMM or RRMM who had reached the plateau of steady disease (SD) or better with bortezomib‐based regimens from April 2018 to April 2020 were observed consecutively. The diagnostic criteria for symptomatic myeloma were defined by the International Myeloma Working Group (IMWG).^15^ Patients diagnosed with plasma cell leukemia and solitary plasmacytoma were excluded. And patients who received autologous stem cell transplantation (ASCT) were also excluded. Finally, 71 patients who received at least two cycles of ixazomib were included in this study. Patients with any of del(17p), t(4;14), gain(1q21), or t(14;16) at diagnosis were defined as a high‐risk genetic cohort. Clinical data before bortezomib‐based therapy were collected retrospectively. Maintenance therapy was started within 60 days of the completion of the induction therapy. Approval of this study was obtained from the Ethics Committee of Beijing Chao‐yang Hospital. Written informed consents were obtained from all patients.

First, we compared the overall response rate (ORR, partial response (PR), or better) and deep response rate (very good PR or better (≥VGPR)) before and after ixazomib‐based maintenance. Then we observed the PFS in groups of NDMM and RRMM respectively and made subgroup analyses among different responses during the induction phase and different changes of responses (deepened, maintained, and progressive) during maintenance therapy. Moreover, we attempted to confirm the independent indicators by multivariate analyses. Furthermore, we assessed the safety and tolerability of ixazomib, including the occurrence rate of common adverse events (AEs) and severe AEs, and the transformation of bortezomib‐emergent PN during the ixazomib maintenance phase.

### 2.2 Treatment

During the induction phase, bortezomib‐based therapies were administered. Bortezomib (1.3 mg/m^2^) was subcutaneously injected on days 1, 4, 8, and 11 of the 21‐day cycle along with standard doses of dexamethasone (20 mg/day) at the same time. The third drug might be liposomal doxorubicin (25 mg/m^2^, intravenous injection, day 4), cyclophosphamide (300 mg/m^2^, intravenous injection, days 1–4), or lenalidomide (25 mg, days 1–21, doses were modulated according to patient's creatinine clearance rate (Clcr)), respectively. The number of patients who received bortezomib at first, second, third, and at least fourth‐line therapy was 37, 19, 3, and 12, respectively.

Ixazomib (4 mg) was administered orally on days 1, 8, and 15, in a 28‐day cycle in the maintenance phase. The regimens included: ixazomib monotherapy (I), Id (ixazomib + dexamethasone 20 mg on days 1, 8, 15, and 22 orally), and IRd (Id+ lenalidomide 25 mg, days 1–21, doses were modulated based on patient's Clcr). Patients discontinued the study for progressive disease (PD) or unacceptable toxicities that were not controlled by lengthening the intermittent period.

### 2.3 Outcome and safety evaluation

Myeloma response criteria was defined according to published IMWG guidelines.^16^ Responses were assessed at the end of induction therapy, and every two cycles during maintenance therapy until PD. Plateau phase was defined as stable serum and urine M‐protein values (within 25% above or below the value at the time the response was assessed) maintaining for at least 3 months with SD or better response; without symptoms of myeloma or blood transfusion.^17^

In either NDMM or RRMM group, PFS of total PI therapy (PFS (total PI)) was measured from the time of patients receiving bortezomib, and PFS of ixazomib (PFS (Ixa)) was defined as the initiation of ixazomib, and both of them were cut off on the date of PD or death due to any cause.

Safety and tolerability were monitored throughout maintenance therapy by recording the incidence, severity, and type of any AEs and graded using the National Cancer Institute Common Terminology Criteria version 4.0.

### 2.4 Statistical analyses

Statistical analyses were performed using the SPSS (version 24.0) software package and *p* < 0.05 were considered statistically significant. Response rates were compared using Wilcoxon tests. Survival probabilities were estimated using the Kaplan–Meier method and the Log‐Rank test was used for univariate comparison. Individual risk factors associated with PFS in univariate analyses (*p *< 0.10) were tested in Cox multivariate regression analyses in which hazard ratios (HR) and 95% confidence intervals (CI) were estimated. All tests were two‐sided, with the type 1 error rate fixed at α = 0.05. All other data were summarized descriptively.

## 3 RESULTS

### 3.1 Patients characteristics and exposure

A total of 71 patients met the predetermined criteria for inclusion in this study including 37 NDMM and 34 RRMM (19 first relapsed, 3 second relapsed, and 12 relapsed ≥3 lines). The median age was 63 (41–80) years and 60.6% of patients were male. Baseline clinical characteristics were summarized in Table 1.

**TABLE 1:** Baseline clinical characteristics of the patients

	Overall (*N *= 71)
Median age, years (range)	63 (41–80)
Age ≥65 years, *n* (%)	25 (35.2)
Male, *n* (%)	43 (60.6)
NDMM, *n* (%)	37 (52.1)
RRMM, *n* (%)	34 (47.9)
ECOG PS score, *n* (%)	
0–1	22 (31.0)
2	39 (54.9)
≥3	10 (14.1)
ISS disease stage at diagnosis, *n* (%)	
I	6 (8.5)
Ⅱ	29 (40.8)
Ⅲ	36 (50.7)
MM subtype, *n* (%)	
IgG	31 (43.7)
IgA	20 (28.2)
Light chain	17 (23.9)
Others^a^	3 (4.2)
Creatinine clearance, ml/min	
Median (range)	61.4 (6–189)
≥90, *n* (%)	16 (22.5)
60 to <90, *n* (%)	21 (29.6)
30 to <60, *n* (%)	18 (25.4)
<30, *n* (%)	16 (22.5)
LDH, median (range), U/L	169.8 (50.8–899.7)
Extramedullary plasmacytoma, *n* (%)	27 (38.0)
Cytogenetic features, *n* (%)	
Standard‐risk cytogenetic abnormalities	28 (39.4)
High‐risk cytogenetic abnormalities^b^	31 (43.7)
Data not available	12 (16.9)
Bortezomib‐emergent PN of grade ≥1, *n* (%)	
Yes	65 (91.5)

Abbreviations: ECOG PS, Eastern Cooperative Oncology Group performance status; ISS, International Staging System; LDH, lactate dehydrogenase; NDMM, newly diagnosed multiple myeloma; PN, peripheral neuritis; RRMM refractory/relapsed multiple myeloma.

^a^ Others were defined as IgD, IgE, and non‐secretory MM.

^b^ High‐risk cytogenetic abnormalities were defined as any of del(17), gain(1q21), t(4;14) and t(14;16).

With a median of 26.5 (7.1–56.8) months to follow up to 1 April 2021, all patients received a median of nine cycles (range 6–14) of bortezomib and a median of six cycles (range 2–25) of ixazomib subsequently. IRd regimen was administered in 17 patients out of the high‐risk cohort. Treatment exposure and reasons for discontinuation were shown in Table 2. Within 15 patients (21.1%) completed ≥12 cycles of ixazomib, three (4.2%) patients had a duration of up to 20 cycles. At the end of the latest follow‐up, 20 cases (28.2%) remained on maintenance treatment with a median of 13.5 courses (4–25) of ixazomib. The most frequently reported reason for discontinuation was individual preference (28.2%). No permanent drug interruptions or death were observed related to AE throughout the ixazomib maintenance phase.

**TABLE 2:** Treatment exposure and reasons for discontinuation (*N *= 71)

Median cycles of bortezomib received (range)	9 (6–14)
BD (bortezomib and dexamethasone), *n* (%)	30 (42.3%)
BCD (bortezomib, dexamethasone and cyclophosphamide), *n* (%)	27 (38.0%)
BDD (bortezomib, dexamethasone and liposomal doxorubicin), *n* (%)	10 (14.1%)
BRD (bortezomib, dexamethasone and lenalidomide), *n* (%)	4 (5.6%)
Median cycles of ixazomib received (range)	6 (2–25)
I (ixazomib), *n* (%)	11 (15.5%)
Id (ixazomib and dexamethasone), *n* (%)	43 (60.6%)
IRd (ixazomib, dexamethasone and lenalidomide), *n* (%)	17 (23.9%)
Cycles of ixazomib received, *n* (%)	
≥6	42 (59.2%)
≥8	28 (39.4%)
≥10	22 (31.0%)
Median cycles of bortezomib plus ixazomib received (range)	15 (8–31)
Patients remaining on treatment, *n* (%)	20 (28.2%)
Reasons for discontinuation, *n* (%)	
Adverse events	8 (11.3%)
Disease progression	16 (22.5%)
Economics	7 (9.9%)
Preference	20 (28.2%)

### 3.2 Responses

**FIGURE 1:**
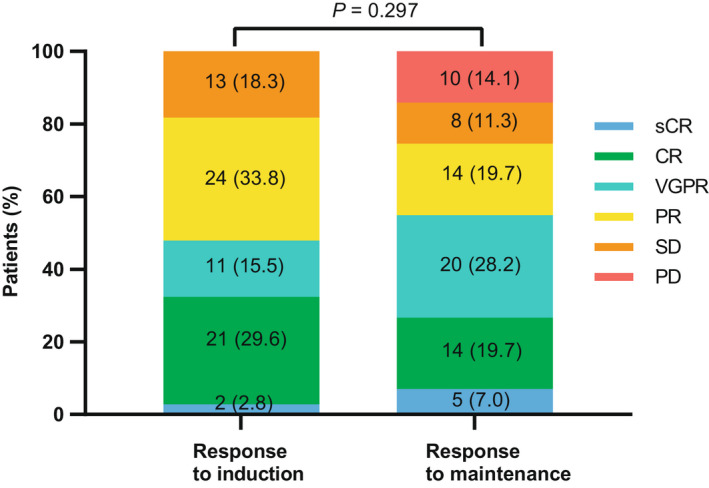
Changes in response rates during induction and maintenance in total population (*N* = 71)

During the induction phase, the last confirmed ORR was 81.7% (58/71), including 34 (47.9%) patients with ≥VGPR, 21 (29.6%) with complete response (CR), and two achieving stringent CR (sCR) (2.8%). After the transition to ixazomib, the best confirmed ORR was 74.6% (53/71), including 39 (54.9%) patients ≥VGPR, 14 (19.7%) CR and 5 sCR (7.0%). Eighteen (25.4%) patients exhibited a deepened response after the transition to ixazomib, including four patients transitioning from CR to sCR, eight transitioning from PR to VGPR, five transitioning from SD to PR, and one transitioning from SD to VGPR (Figure 1). There were numerically more ≥VGPRs after maintenance therapy, but the differences were not significant (*p *= 0.297).

Among the 31 patients from the high‐risk genetic cohort, eight (25.8%) patients exhibited a deepened response including six patients transitioning from PR to VGPR, and two from SD to PR. The ORR, ≥VGPR, and CR rate for the high‐risk genetic subgroup was comparable with the overall population in either the induction phase or maintenance phase (*p *= 0.317 and *p *= 0.424, respectively).

In the NDMM subgroup, the ORR was transformed from 100% to 82.4% after transitioning to ixazomib, and ≥VGPR rate increased from 62.2% to 70.3% as well. Of these, eight patients (21.6%) exhibited responses deepened, 23 patients (62.2%) maintained, and six patients (16.2%) progressed. Meanwhile, in the RRMM cohort, the ORR maintained 61.8%, while ≥VGPR rate increased from 32.4% to 38.2%. Of these, ten patients (29.4%) exhibited responses deepened, 11 patients (32.4%) maintained, and 13 patients (38.2%) progressed.

### 3.3 Survival analyses

At the latest follow‐up, 17 patients were dead. Twelve patients died of PD from the RRMM cohort and five died of non‐PD from the NDMM cohort (one case of pneumonia, one case of cardiogenic shock, and three cases of cerebrovascular accident).

With 26.5 months median follow‐up, the median PFS (total PI) and PFS (Ixa) were 24.9 and 15.3 months within the total population, respectively. However, we separately analyzed the PFS of NDMM and RRMM as follows since these two entities were different.

### 3.4 Deepened responses indicated PFS prolongation in NDMM cohort

**FIGURE 2:**
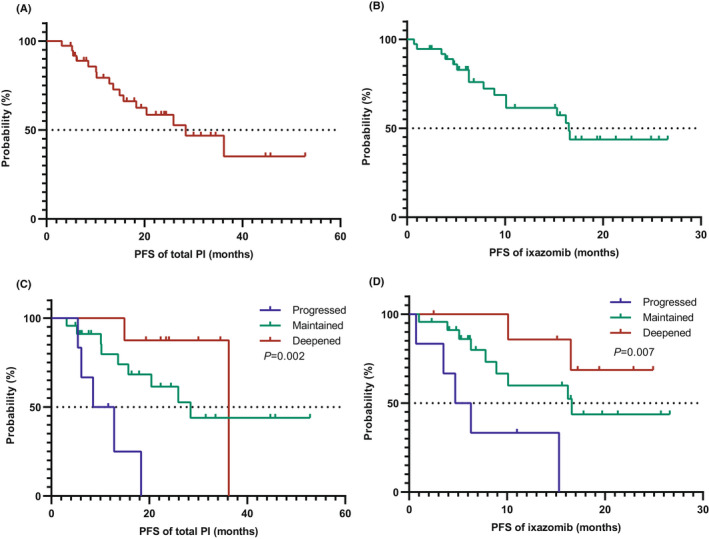
Kaplan‐Meier analyses of PFS in the NDMM cohort (*N* = 37). (A) PFS of total PI therapy (from initiation of bortezomib). (B) PFS of ixazomib (from initiation of ixazomib). (C) PFS of total PI therapy among different response changes (deepened, maintained, and progressed) during maintenance. (D) PFS of ixazomib among different response changes (deepened, maintained, and progressed) during maintenance

In the NDMM cohort, the median estimated PFS (total PI) was 28.4 months (14.8–42.1) with a median follow‐up of 26.8 months (7.1–56.8), and the 1‐year and 2‐year PFS (total PI) rates were 76.0% and 58.5%, respectively (Figure 2A). The median estimated PFS (Ixa) was 16.5 months (14.7–18.3), with a 1‐year and 2‐year PFS (Ixa) rate of 61.5% and 43.3%, respectively (Figure 2B).

Depending on responses transformation (deepened, maintained, and progressed) after ixazomib maintenance, the median estimated PFS (total PI) was 36.2, 28.4, and 8.5 months, and the median estimated PFS (Ixa) was not reached (NR), 16.6, and 4.7 months, respectively. There were significant differences on PFS (total PI) (*p* = 0.002) (Figure 2C) and PFS (Ixa) (*p* = 0.007) (Figure 2D) among three groups.

However, a significant difference was not observed between standard and high‐risk cytogenetic patients (*p *= 0.703 and *p *= 0.883), young patients (<65 years) and elderly patients (≥65 years) (*p *= 0.378 and *p *= 0.246), and among the three ISS categories (*p *= 0.636 and *p *= 0.809) in terms of PFS (total PI) and PFS (Ixa), respectively.

### 3.5 VGPR or better during induction brought benefit to PFS in RRMM cohort

**FIGURE 3:**
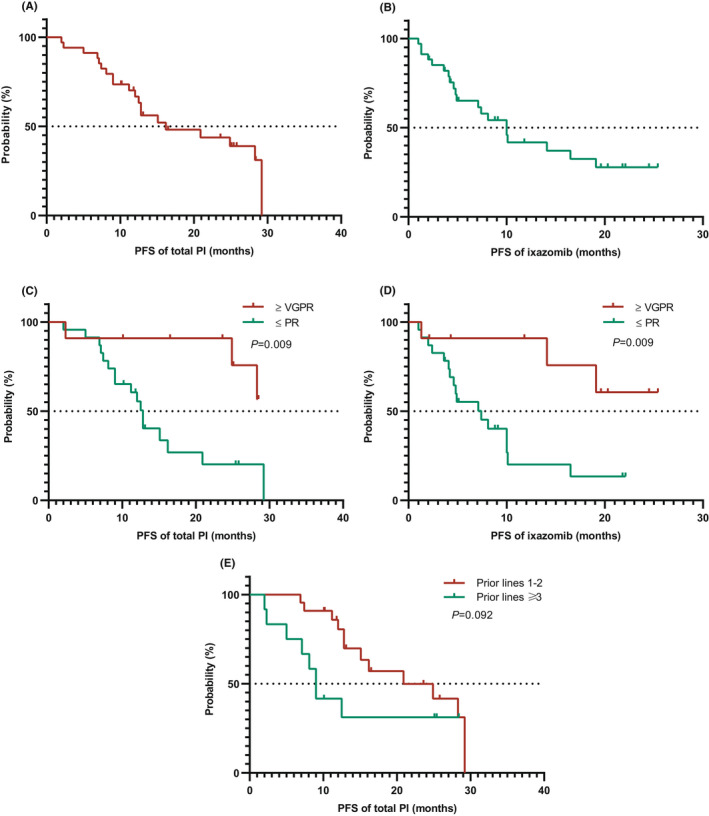
Kaplan‐Meier analyses of PFS in the RRMM cohort (*N* = 34). (A) PFS of total PI therapy (from initiation of bortezomib). (B) PFS of ixazomib (from initiation of ixazomib). (C) PFS of total PI therapy in patients with ≥VGPR or ≤PR during induction. (D) PFS of ixazomib in patients with ≥VGPR or ≤PR during induction. (E) PFS of total PI therapy according to prior lines of treatment (1–2 vs. ≥3).

In the RRMM cohort, the median estimated PFS (total PI) was 16.2 months (4.4–28.1) with a median follow‐up of 25.8 months (10.1–41.5), and the 1‐year and 2‐year PFS (total PI) rates of 66.7% and 38.9%, respectively (Figure 3A). The median PFS (Ixa) was 10.0 months (6.9–13.1), with 1‐year and 2‐year PFS (Ixa) rates of 41.7% and 27.8%, respectively (Figure 3B).

VGPR or better during induction brought benefit to both PFS (total PI) (median NR vs. 12.7 months, *p *= 0.009, 2‐year PFS rate 75.8% vs. 20.2%) (Figure 3C) and PFS (Ixa) (median NR vs. 7.43 months, *p *= 0.009) (Figure 3D). Moreover, in terms of PFS (total PI), a separate trend was observed between heavily treated patients (prior lines ≥3) and those without (prior lines 1 and 2) (median 20.9 vs. 9.0 months, *p *= 0.092) (Figure 3E).

However, a significant difference was not observed between standard and high‐risk cytogenetic patients (*p *= 0.863 and *p *= 0.701), young patients (<65 years) and elderly patients (≥65 years) (*p *= 0.932 and *p *= 0.540), and among the three ISS categories (*p *= 0.760 and *p *= 0.791) in terms of PFS (total PI) and PFS (Ixa), respectively.

### 3.6 Multivariate analyses for survival

Univariate and multivariate analyses of PFS were performed on the clinical parameters including age, LDH, ISS, cytogenetic risk, disease status (NDMM vs. RRMM), response during the induction phase (≥VGPR vs. ≤PR), and responses transformation during ixazomib maintenance (deepened vs. not). Worth mentioning, patients of responses maintained, and progressed were combined as the group of responses not deepened to compare with those of responses deepened. Because of the overlap between ISS and Clcr in terms of renal function, Clcr was not included in the univariate analyses.

In univariate analyses, NDMM, ≥VGPR during induction, responses deepened after maintenance and without extramedullary plasmacytoma were associated with prolonged PFS (total PI) (Table 3); except for NDMM, the above variables were also associated with prolonged PFS (Ixa) (Table 4). Then multivariate analyses were performed subsequently with the parameters associated with PFS in the univariate analyses.

**TABLE 3:** Univariate and multivariate analyses of covariates affecting PFS (total PI) (*N *= 71).

Covariates	Univariate			Multivariate		
	HR	95% CI	*p* value	HR	95% CI	*p* value
Age (y)						
≥65 vs. <65	1.152	0.587–2.258	0.681			
Gender						
Male vs. female	1.274	0.633–2.563	0.497			
MM status						
RRMM vs. NDMM	1.784	0.904–3.518	0.095	1.107	0.473–2.185	0.966
ISS stage						
Ⅲ vs. I–Ⅱ	1.264	0.642–2.490	0.498			
LDH (U/L)						
≥ 250 vs. <250	1.332	0.469–3.777	0.590			
Extramedullary plasmacytoma						
Yes vs. no	2.248	1.154–4.379	0.017^*^	3.070	1.406–6.702	0.005^**^
Cytogenetic risk^a^						
High vs. standard	1.224	0.612–2.445	0.568			
Response during induction						
≥VGPR vs. ≤PR	0.377	0.187–0.762	0.007^**^	0.133	0.054–0.331	<0.001^***^
Responses deepened						
Yes vs. no^b^	0.387	0.160–0.934	0.035^*^	0.195	0.073–0.525	0.001^**^

Abbreviations: ISS, International Staging System; LDH, lactate dehydrogenase; NDMM, newly diagnosed multiple myeloma; RRMM, refractory/relapsed multiple myeloma.

^a^ High‐risk cytogenetics weas defined as any of del(17), gain(1q21), t(4;14) and t(14;16).

^b^ No responses deepened included patients of responses maintained and responses progressed.

* *p* < 0.05, ^**^ *p* < 0.01, ^***^ *p* < 0.001.

**TABLE 4:** Univariate and multivariate analyses of covariates affecting PFS (Ixa) (*N *= 71)

Covariates	Univariate			Multivariate		
	HR	95% CI	*p* value	HR	95% CI	*p* value
Age (years)						
≥65 vs. <65	1.101	0.562–2.156	0.779			
Gender						
Male vs. female	0.969	0.489–1.921	0.929			
MM status						
RRMM vs. NDMM	1.663	0.860–3.216	0.130			
ISS stage						
Ⅲ vs. I–Ⅱ	1.022	0.527–1.980	0.949			
LDH (U/L)						
≥250 vs. <250	1.279	0.452–3.622	0.643			
Extramedullary plasmacytoma						
Yes vs. no	2.290	1.185–4.424	0.014^*^	2.152	1.082–4.280	0.029^*^
Cytogenetic risk^a^						
High vs. standard	1.062	0.535–2.105	0.865			
Response during induction						
≥VGPR vs. ≤PR	0.365	0.180–0.741	0.005^**^	0.218	0.102–0.463	<0.001^***^
Responses deepened						
Yes vs. no^b^	0.390	0.161–0.943	0.037^*^	0.270	0.104–0.700	0.007^**^

Abbreviations: ISS, International Staging System; LDH, lactate dehydrogenase; NDMM, newly diagnosed multiple myeloma; RRMM, refractory/relapsed multiple myeloma.

^a^ High‐risk cytogenetics weas defined as any of del(17), gain(1q21), t(4;14) and t(14;16).

^b^ No responses deepened included patients of responses maintained and responses progressed.

* *p* < 0.05, ^**^ *p* < 0.01, ^***^ *p* < 0.001.

As such, responses deepened after maintenance was also identified as an independent indicator of both PFS (total PI) (HR: 0.195, *p *= 0.001) and PFS (Ixa) (HR: 0.270, *p *= 0.007). Moreover, ≥VGPR during induction was identified as an independent indicator of both PFS (total PI) (HR: 0.133, *p *< 0.001) and PFS (Ixa) (HR: 0.218, *p *< 0.001). Additionally, extramedullary plasmacytoma was an independent risk factor for PFS, with the HR value of 3.070 (*p *= 0.005) in PFS (total PI) and 2.152 (*p *= 0.029) in PFS (Ixa), respectively.

### 3.7 Safety profile and discontinuation

AEs and discontinuation during the maintenance phase were shown in Table 5. The most common observed hematological AE was neutropenia (12.7%), while diarrhea (23.9%) was the most common non‐hematological AE. Grade 3/4 AEs which led to discontinuation were reported in eight (11.2%) patients, including diarrhea, vomit, and neutropenia in 5.6%, 4.2%, and 1.4%, respectively.

**TABLE 5:** Safety analyses of 71 patients during the maintenance phase (*N *= 71)

	Any grade	Grade 3/4
Non‐hematologic AEs, *n* (%)		
Diarrhea	17 (23.9%)	4 (5.6%)
Vomit and nausea	10 (14.1%)	3 (4.2%)
Rash eruptions	9 (12.7%)	—
Infection	4 (5.6%)	—
Fatigue	3 (4.2%)	—
Constipation	3 (2.8%)	—
Herpes zoster	2 (2.8%)	—
Liver dysfunction	2 (2.8%)	—
Hematologic, *n* (%)		
Neutropenia	9 (12.7%)	1 (1.4%)
Thrombocytopenia	4 (5.6%)	—
Anemia	3 (4.2%)	—

**FIGURE 4:**
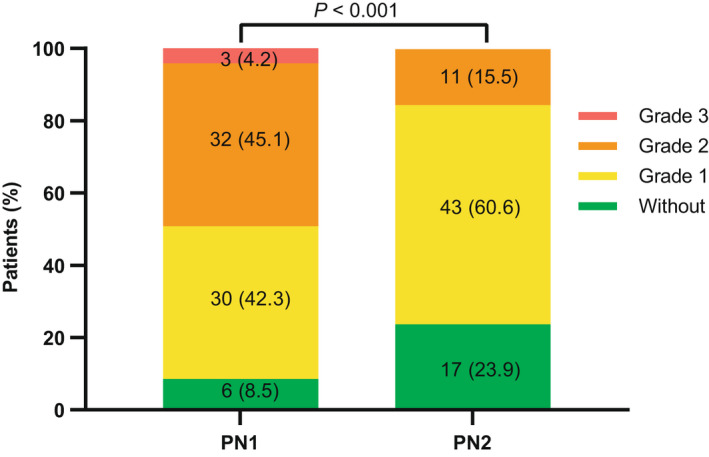
Changes in grades of peripheral neuritis (PN) between induction phase (PN1) and maintenance phase (PN2) in total population (*N* = 71)

Of note, bortezomib‐emergent PN (BiPN) was the prevalent toxicity during induction, with 91.5% of any grade and 4.2% of grade 3. At the time of cutoff for maintenance, PN had resolved in 11 patients; among the 54 ongoing cases (76.1%), three cases (4.2%) decreased from grade 3 to 2, and 24 cases (33.8%) from grade 2 to 1. Meanwhile, there was no new‐onset case of any grade PN. A statistically significant difference was observed in comparison (*p *< 0.001) (Figure 4).

## 4 DISCUSSION

This real‐world study reported the use of the ixazomib‐based regimen as maintenance therapy in 71 MM patients who were plateaued by bortezomib‐based therapy but not undergoing transplantation. With deepening responses but worsening PN, the ixazomib‐based regimen exhibited a good therapeutic efficacy with feasible tolerance. In addition, responses deepened during maintenance and ≥VGPR during induction have demonstrated the association with prolongation of PFS.

Several clinical trials demonstrated that ixazomib‐based regimens were effective in NDMM,^13^ RRMM,^10^, ^11^ and MM maintenance therapy after transplantation.^12^ However, there were limited findings from the real‐world study of ixazomib in MM patients not undergoing transplantation. Unlike strictly selected in clinical trials, our study population was older (median 63 years old), with worse ECOG (PS ≥2 of 69%), more advanced ISS stage (ISS Ⅲ of 50.7%), and more terminal renal function (Clcr <60 ml/min of 47.9%). The discrepancy of baseline characteristics and treatment exposure might lead to disparities in outcome. In our NDMM cohort, the ORR was 86.5% including a 70.3% ≥VGPR rate and a 35.1% CR rate in comparison to 94%, 63%, and 35% respectively in an integrated analysis of four early‐phase trials about single‐agent ixazomib maintenance therapy in NDMM patients. We found that patients achieving ≥VGPR experienced significantly longer PFS than patients achieving ≤PR from prior therapies. Similarly, deep efficacy can be translated into survival benefits according to a clinical trial of ixazomib.^18^

Moreover, we found a total of 25.4% deepening their responses after the transition to ixazomib, including 21.6% in the NDMM subgroup. Similar observations of responses deepened were reported in several clinical trials about transplant‐ineligible NDMM patients, such as 14.6% in TOURMALINE‐MM4,^18^ 23% in a phase Ⅰ/Ⅱ study investigating IMP induction followed by ixazomib as maintenance,^19^ and 10% in a phase Ⅱ study of ITd induction followed by ixazomib as maintenance.^20^ Responses improvements might result from the continued decline of the plasma cell clone and ongoing clearance of the M‐protein in those patients with sensitive, less proliferative, and more mature clones.^21^ Furthermore, in our study, patients with responses deepened after maintenance therapy were confirmed independently associated with prolongation of PFS (Ixa) with a hazard ratio of 0.270 (*p *= 0.007) compared to those without. Similarly, a recent phase Ⅲ study of TOURMALINE‐MM3 demonstrated that PFS was prolonged among those who had deepened responses regardless of treatment arm (HR = 0.252, *p *< 0.001).^21^

A median of 15.3 months of PFS was observed from the initiation of ixazomib in the total population, with 16.5 months in the NDMM subgroup. Similar observations were reported in ixazomib monotherapy maintenance studies, such as TOURMALINE‐MM4 with a median PFS of 17.4 months,^18^ and 14.3 months in single ixazomib maintenance post‐ITd induction of RRMM patients.^22^ Differently, regimens of IRd (23.9%) and Id (60.6%) were included in our cohort, besides 15.5% of single ixazomib. This may be correlated to the high proportion (43.7%) of the high‐risk entity in our study. Of these, 17 patients received the IRd regimen, and 12 cases of the remaining high‐risk cohort received the Id regimen. Maybe the discrepancy in treatment resulted in a comparable outcome between the high‐risk cohort and the overall population.

In our study, ixazomib was well tolerated as 21.1% of patients had a duration of at least 12 cycles, and the ongoing 20 patients had a median of 13.5 cycles at the last follow‐up, further supporting the feasibility and clinical value of prolonged ixazomib in the real‐world setting. Compared to TOURMALINE‐MM4, we found similar drug‐related events in terms of any grade (63.4% vs. 66.7%), grade ≥3 (11.3% vs. 17.8%), and drug‐related discontinuation (11.3% vs. 12.9%). Gastrointestinal effects, the most common non‐hematological toxicity (40.8%), could be relieved after actively supportive treatment in our study. Notably, 16 cases with terminal renal function (≤30 ml/min Clcr) were included in our study with achieving improvements in renal function and hematological remission after ixazomib maintenance. Without the availability of 3 mg ixazomib, a median of 12 cycles of ixazomib‐based therapy was administered in these patients by lengthening the intermittent period. In these patients, the ORR and ≥VGPR rate was 62.5% and 50%, respectively, concomitant with a 31.3% rate of responses deepening. Hence, in the real‐world setting, ixazomib could be a preferable option for long‐term treatment in patients with renal dysfunction.

Bortezomib‐related PN had been the major toxicity during induction, preventing a substantial number of patients from starting maintenance. As in the HOVON‐65/GMMG‐HD4 study, the incidence of PN was increased from 40% to 45% after a two‐year duration of biweekly bortezomib.^8^ However, in our cohort, the incidence of PN changed from 91.5% to 76.1% after ixazomib maintenance, without new PN occurring. A similar observation was reported in another real‐world study of IRd regimen in RRMM.^23^ It is encouraging for the PN intolerant patients as switching to ixazomib is feasible.

As a retrospective study, some limitations should be considered. One main limitation is that the relatively small number of patients in a single institution. Another limitation is the lack of a comparison with other established regimens. Additionally, the heterogeneity in terms of both induction and maintenance regimens should be considered. Hence, further multi‐center and larger prospective studies are needed to verify the results. Nevertheless, this study demonstrated the efficacy and feasibility of ixazomib‐based maintenance after induction of bortezomib‐based in MM patients not undergoing transplantation in real‐world practice. Different from bortezomib, this weekly oral PI brings more convenience for patients without worrying about the distance to the treatment center for injectable therapies, and the concomitant loss of days of work, which confirmed its long‐term administration in real life. In addition, in the context of the pandemic spread of COVID‐19, which greatly altered the therapeutic style of MM patients, the oral weekly regimen could reduce the visits to the hospital, and decrease the exposure to infection.

In conclusion, we reported the data from the real‐life study on the effectiveness and safety profile of ixazomib‐based maintenance therapy in MM not undergoing transplantation. And the larger sample size, longer follow‐up, and case‐controlled clinical study of ixazomib‐based therapy for MM patients also deserved to be performed in the future.

## AUTHOR CONTRIBUTION STATEMENT

MS and JJZ performed the research; XL and YPZ designed the research; RT, YHW, XKZ, and SBF contributed to data collection; MS analyzed the data; MS wrote the paper; XL and ZXH revised the paper.

## CONFLICT OF INTEREST

The authors have no competing interests.

## ETHICAL APPROVAL

Approval of this study was obtained from the Ethics Committee of Beijing Chao‐yang Hospital. Written informed consents were obtained from all patients.

## Data Availability

The data that support the findings of this study are available on request from the corresponding author. The data are not publicly available due to privacy or ethical restrictions.
